# Prevalence and Influencing Risk Factors of Eczema Among Preschool Children in Hail City

**DOI:** 10.7759/cureus.32723

**Published:** 2022-12-20

**Authors:** Fawwaz F Alshammrie, Sarah K Albarrak, Atheer A Alhuthaili, Sara A Alakash, Mohammed H Al Mansour, Marwa R Gammash

**Affiliations:** 1 Dermatology, University of Hail, Hail, SAU; 2 Medicine and Surgery, University of Hail College of Medicine, Hail, SAU; 3 Medicine and Surgery, Imam Abdulrahman Bin Faisal University, Dammam, SAU; 4 Otolaryngology, Najran General Hospital, Najran, SAU; 5 Dermatology, East Jeddah Hospital, Jeddah, SAU

**Keywords:** hail, children, risk factors, prevalence, atopic dermatitis, eczema

## Abstract

Background

Eczema is a common inflammatory skin disorder in the pediatric population. Although eczema is a significant public health issue with negative impacts on quality of life, studies looking at the prevalence and risk factors among preschool-aged children in Saudi Arabia are limited.

Methods

A descriptive, cross-sectional, online-based study was conducted among parents of preschool children in Hail City, Saudi Arabia, between July 2022 and September 2022. The self-administered, pre-tested questionnaire was distributed on social media networks for data gathering, which was subsequently analyzed using Statistical Package for the Social Sciences (SPSS, IBM Corp., Armonk, NY) software.

Results

Among 964 preschool children, 54.5% of the children have been diagnosed with eczema. A total of 152 (60.1%) male children had eczema compared to 231 (59.8%) female children, while 142 (43.7%) parents had children of both genders diagnosed with eczema (P = 0.001). Multivariate logistic regression showed that male gender (odds ratio [OR] = 1.41, 95% confidence interval = 1.16-1.71), smoking in the house (OR = 1.85, 95%CI = 1.37-2.50), presence of mold or dampness in the house while the mother was pregnant (OR = 1.95, 95%CI = 1.17-3.24), house renovation during the mother's pregnancy (OR = 1.54, 95%CI = 1.01-2.34), use of an air conditioner (OR = 1.57, 95%CI = 1.07-2.30), and dry skin (OR = 5.83, 95%CI = 4.27-7.96) were significant predictors for the development of eczema.

Conclusion

The high prevalence of eczema among preschool-aged children in Hail indicates the need for parents to take action at the household level and beyond to successfully lower the risk of eczema development in the region.

## Introduction

Eczema is an umbrella term referring to multiple types of dermatitis that can be either endogenous, such as atopic dermatitis (AD), or exogenous, such as allergic and irritant contact dermatitis. Non-dermatologists and the general public frequently use the term "eczema" to describe AD. In addition to AD, eczema encompasses other conditions such as contact dermatitis, nummular eczema, and seborrheic dermatitis, all of which manifest as itching and erythema and can be distinguished by their etiologies, morphologies, or distribution patterns [1]. In children, AD (or atopic eczema) is the most prevalent type of eczema. The primary manifestation of AD is a chronic, relapsing rash with itching that often begins in infancy and typically gets better later in childhood in many patients. Many people with atopic eczema do not exhibit an IgE reaction to common allergens. However, the terms "atopic eczema" and "atopic dermatitis" are still frequently employed to describe poorly defined inflammatory skin conditions with surface changes, a preference for flexures, and a family or personal history of allergic rhinitis or asthma [2]. The itching associated with eczema can disrupt children's sleep patterns and, in more severe cases, potentially impair physical and mental development [3].

Eczema is the most prevalent childhood inflammatory skin condition, affecting 2-20% of children worldwide and resulting in significant morbidity and cost burden [4,5]. The incidence of eczema has markedly increased recently, but the prevalence continues to vary in different countries [2,6,7]. The causes of this large variance in eczema prevalence are not known. Eczema is a complex skin condition that can vary in severity due to genetics, environmental factors, and cultural exposures [3,6,8,9].

In Southern California, a study was performed on 3302 children of different ethnicities, including Hispanic and non-Hispanic, who were aged five to seven years. The study revealed that 16.9% of children were diagnosed with eczema, and the prevalence was significantly higher in non-Hispanic children [6]. A cross-sectional study was carried out by Bornehag et al. on 10,851 children in Sweden. Data were collected by a questionnaire distributed to the parents, which showed that 18.7% of children had eczema in the last 12 months [10]. An Italian prospective multicenter cohort study was conducted to assess perinatal risk factors for eczema; subsequently, it showed that a family history of eczema and/or asthma was linked to a higher risk of eczema. On the other hand, there was no relationship identified between breastfeeding or smoking and the risk of eczema [11].

Eczema is a major public health problem worldwide. Monitoring the prevalence and exacerbating factors for this disease in different countries will enable the identification of triggers, improve quality of life, and reduce the cost burden. Studies have shown that eczema is preventable [2], and management of risk factors or triggers should be a public health initiative. This is the first eczema study of preschool-aged children in Hail City. The goal is the assessment of eczema prevalence and the identification of risk factors. Subsequently, implement the intervention with health education programs to reduce triggering risk factors.

## Materials and methods

A descriptive cross-sectional study was conducted in Hail City between July 2022 and September 2022 to assess the prevalence rate of eczema and its influencing risk factors among preschool children. The target subjects were parents of preschool children in Hail, Saudi Arabia, of both genders and different nationalities. The data were collected using a self-report questionnaire, which was provided via Google Forms in an electronic format. The Statistical Package for the Social Sciences (SPSS, IBM Corp., Armonk, NY) program was used to perform the statistical analysis after importing the data into Microsoft Excel (Microsoft® Corp., Redmond, WA).

Inclusion and exclusion criteria

Parents of preschool children of both genders, ages two to eight, living in Hail, Saudi Arabia, regardless of nationality, were included. Parents with children not living in Hail, children outside of the age range, and incomplete questionnaires were all excluded from the study.

Sampling method and sample size calculation

The study sample size was 377 parents as estimated using a Raosoft® Sample Size Calculator, which is representative of Hail's population of 19,042 with a 5% margin of error, a 95% level of confidence, and a 50% response distribution. For the purpose of avoiding possible exclusions and increasing power and validity, we aimed to get a larger sample size. The non-probability convenience sampling method was employed.

Data collection instruments and procedures

This study used a pretested, self-report, web-based survey to collect data. Informed consent was first acquired from all the parents who agreed to participate after explaining the aim of the study on the first page of the survey. On several social media networks, including WhatsApp, Facebook, Twitter, and Telegram, a Google Form survey (Google LLC, Mountain View, CA, USA) was distributed electronically for data gathering. A group of data collectors from various locations in Hail City has been enlisted to gather the data from their respective areas to increase the number of participants in this study. The questionnaire was divided into the following sections: (1) bio-demographic data; (2) eczema history-related questions; (3) health issues affecting the kid and his/her family, such as wheezy chest, asthma, pneumonia, food allergies, allergic rhinitis, and other related conditions; (4) the child's living area, such as the type of residence, furnishings, ventilation, and smoke elimination; (5) environmental factors assessment, such as the presence of pets, plants at home, household smokers, and cleaning regularity; and (6) moisturizer usage. The third, fourth, and fifth sections were adapted from Shi et al. [3]. The remaining sections were prepared and added following an extensive review of the literature on topics related to eczema with similar objectives. Minor adjustments were made in light of certain circumstances in Hail. Dermatology professionals reviewed the final survey to ensure it was clear and simple, as well as relevant to the study's objectives. A pilot study of 20 participants was conducted to ensure the coherence and wording of the final survey, and the included participants were excluded from the sample. The questionnaire was translated into Arabic using accredited translation software so that respondents could select between the Arabic or English versions according to their preference. In order to prevent the duplication of responses, the link was permitted to receive only a single response from each participant.

Statistical analysis of the data

After the data were extracted, they were revised, coded, and fed to the statistical software IBM SPSS version 22 (SPSS, Inc., Chicago, IL). Two-tailed tests were applied for all statistical analyses. The level of statistical significance was judged to be a P-value less than 0.05. Descriptive analysis based on frequency and percent distribution was done for all variables, including children's bio-demographic data, eczema history, and prevalence of eczema. Crosstabulation was used to assess different environmental, social, and other factors associated with having eczema among the studied children. Pearson's chi-squared test was employed to test relations, and small frequency distributions were tested using the exact probability test. A multiple stepwise logistic regression model was used, including all identified significant determinants in univariate analysis, to detect the most significant predictors associated with having eczema among the children, with the consideration of keeping all other factors constant, and the odds ratio (OR), effect size, and 95% confidence interval (CI) were estimated.

Confidentiality and ethical approval

This study has been reviewed and approved by the Research Ethics Committee (REC) at the University of Hail with the approval number H-2022-285. To maintain participant confidentiality, personal data, including the participant's name, mobile number, and address, were not requested in the questionnaire.

## Results

**Figure 1:**
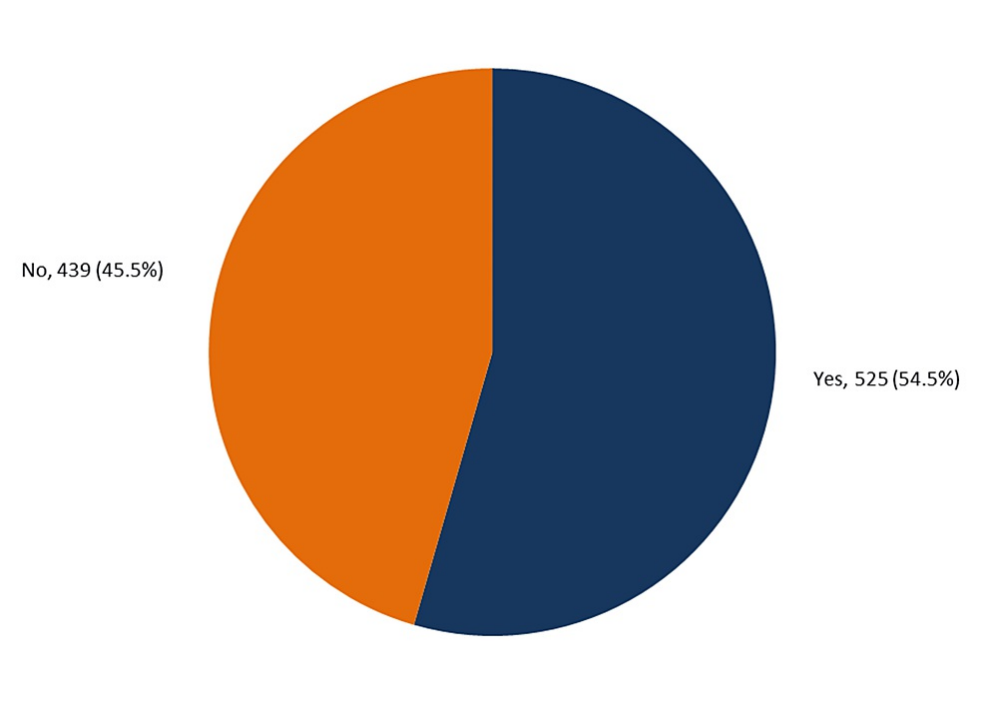
Prevalence of eczema among preschool children in Hail City, Saudi Arabia.

A total of 964 parents of eligible children completed the questionnaire. The children's ages ranged from 2 to 8 years, with a mean age of 4.6 ± 1.2 years. Regarding gender, 40% of respondents had female children and 26.2% had male children, but 33.78% had children of both genders. Five hundred and twenty-five (54.5%) of parents reported having a confirmed eczema-affected child or children, while 439 (45.5%) had not (Figure 1).

The study revealed that age was insignificantly associated with eczema among children (P = 0.187). A total of 60.1% of male children had eczema, compared to 59.8% of female children (P = 0.001). In terms of the past medical history of children with eczema, 75.7% had a history of pneumonia, compared to 75.6% who had a history of allergic rhinitis, and 41.1% of children had no co-morbidities. Moreover, 119 (53.1%) children delivered via cesarean section (CS) had a lower incidence of eczema than those delivered vaginally (P = 0.031). Eczema was diagnosed in 76.5% of children with a family history of eczema versus 39.3% of those without (P = 0.001). Furthermore, 70.4% of children with food allergies had eczema, compared to 43.7% of those without (P = 0.001). In total, 84.5% of children with pityriasis rosea had eczema, compared to 75.5% of those with urticaria and 47.2% of those with no identified dermatological disorders (P = 0.001) (Table 1).

**Table 1:** Distribution of eczema by children bio-demographic data in Hail City, Saudi Arabia. P: Pearson X^2^ test; ^$^exact probability test; *P < 0.05 (significant).

Bio-demographic data	Total		Has a child been diagnosed with eczema?				p-value
			Yes		No		
	No	%	No	%	No	%	
Ages of children							0.187^$^
2–4	345	35.8%	174	50.4%	171	49.6%	
4–6	331	34.3%	187	56.5%	144	43.5%	
6–8	391	40.6%	218	55.8%	173	44.2%	
Gender							0.001*
Male	253	26.2%	152	60.1%	101	39.9%	
Female	386	40.0%	231	59.8%	155	40.2%	
Both	325	33.7%	142	43.7%	183	56.3%	
Co-morbidities							0.001*
Wheezing chest	187	19.4%	139	74.3%	48	25.7%	
Asthma	181	18.8%	131	72.4%	50	27.6%	
Allergic rhinitis	172	17.8%	130	75.6%	42	24.4%	
Pneumonia	70	7.3%	53	75.7%	17	24.3%	
Others	71	7.4%	44	62.0%	27	38.0%	
None	511	53.0%	210	41.1%	301	58.9%	
Mode of children’s delivery							0.031*
NVD	546	56.6%	315	57.7%	231	42.3%	
CS	224	23.2%	119	53.1%	105	46.9%	
Both	194	20.1%	91	46.9%	103	53.1%	
Is there a family history of eczema?							0.001*
Yes	392	40.7%	300	76.5%	92	23.5%	
No	572	59.3%	225	39.3%	347	60.7%	
Does your child/children have a food allergy?							0.001*
Yes	301	31.2%	212	70.4%	89	29.6%	
No	398	41.3%	174	43.7%	224	56.3%	
Don’t know	265	27.5%	139	52.5%	126	47.5%	
Types of allergies children complain							0.001*
Urticaria	94	9.8%	71	75.5%	23	24.5%	
Psoriasis	98	10.2%	67	68.4%	31	31.6%	
Pityriasis rosea	84	8.7%	71	84.5%	13	15.5%	
Others	114	11.8%	69	60.5%	45	39.5%	
Don’t know	644	66.8%	304	47.2%	340	52.8%	

Eczema was diagnosed in 79.1% of children who had an itchy rash that flared up at least every six months, compared to 24.1% of those who did not (P = 0.001). Additionally, 78.9% of children who had an itchy rash at any time in the last 12 months were diagnosed with eczema (P = 0.001). Noting that in 74.5% of children, the itchy rash was completely cleared at any point in the previous 12 months. In children with established diagnoses of eczema, the most commonly reported locations of itchy rash were the anterior aspect of ankle joints (84.3%), eyes (84.3%), the fold of the elbows (83.7%), behind the knees (81.7%), and around the neck (81.7%). Similarly, 82.1% of children with an established diagnosis of eczema had their first presentation of an itchy rash before the age of two years, while 65.8% had their first presentation aged five years or older (p = 0.001) (Table 2).

**Table 2:** Distribution of eczema by children's rash history in Hail City, Saudi Arabia. P: Pearson X^2^ test; ^$^exact probability test; *P < 0.05 (significant).

Rash history	Total		Has a child been diagnosed with eczema?				p-value
			Yes		No		
	No	%	No	%	No	%	
Have your child/children ever had an itchy rash that was coming and going for at least six months?							0.001*
Yes	532	55.2%	421	79.1%	111	20.9%	
No	432	44.8%	104	24.1%	328	75.9%	
Have your child/children had this itchy rash at any time in the last 12 months?							0.001*
Yes	487	81.7%	384	78.9%	103	21.1%	
No	109	18.3%	67	61.5%	42	38.5%	
Has this itchy rash at any time affected any of the following places?							0.001*
Fold of the elbows	246	41.3%	206	83.7%	40	16.3%	
Anterior aspect of the ankle joint	198	33.2%	167	84.3%	31	15.7%	
Under the buttocks	210	35.2%	172	81.9%	38	18.1%	
Behind the knees	235	39.4%	192	81.7%	43	18.3%	
Around the neck	229	38.4%	187	81.7%	42	18.3%	
Ears	171	28.7%	145	84.8%	26	15.2%	
Eyes	108	18.1%	91	84.3%	17	15.7%	
Other regions	176	29.5%	130	73.9%	46	26.1%	
None	39	6.5%	24	61.5%	15	38.5%	
At what age did this itchy rash first occur?							0.001*
<2 years	324	54.4%	266	82.1%	58	17.9%	
2–4 years	158	26.5%	110	69.6%	48	30.4%	
5 years / above	114	19.1%	75	65.8%	39	34.2%	
Has this rash cleared completely at any time during the last 12 months?							0.830
Yes	444	74.5%	335	75.5%	109	24.5%	
No	152	25.5%	116	76.3%	36	23.7%	

The study identified various environmental risk factors that can influence preschoolers' eczema development, including houses with carpets, smoking in houses, smoking during the first year of life, smoking during the mother’s pregnancy, pets in the house during the first year of the child’s life, mold or dampness in the house during the first year of the child’s life or before and during the mother’s pregnancy, house renovations during and before the mother's pregnancy, and the use of air conditioners (all P < 0.05) (Table 3).

**Table 3:** Environmental factors associated with eczema among children in Hail City, Saudi Arabia. P: Pearson X^2^ test; *P < 0.05 (significant).

Environmental factors	Has a child been diagnosed with eczema?				p-value
	Yes		No		
	No	%	No	%	
Are there carpets in your house?	457	87.0%	353	80.4%	0.005*
Does anyone smoke in your house?	288	54.9%	151	34.4%	0.001*
Did anyone smoke in your house during the first year of the child’s/children’s life?	226	43.0%	122	27.8%	0.001*
Did anyone smoke in your house while the mother was pregnant?	213	40.6%	110	25.1%	0.001*
Are there currently flowering plants in your house?	227	43.2%	177	40.3%	0.360
Were there flowering plants in your house during the first year of the child’s /children’s life?	198	37.7%	145	33.0%	0.130
Are there currently pets in your house?	159	30.3%	114	26.0%	0.138
Were there pets in your house during the first year of the child’s/children’s life?	162	30.9%	100	22.8%	0.005*
Was there mold or dampness in your house during the first year of the child’s/children’s life?	82	15.6%	48	10.9%	0.034*
Was there mold or dampness in the house while the mother was pregnant?	74	14.1%	38	8.7%	0.009*
Was there mold or dampness in your house before the mother became pregnant?	80	15.2%	46	10.5%	0.029*
Was your house renovated during the first year of the child’s/children’s life?	169	32.2%	135	30.8%	0.632
Was your house renovated during the mother's pregnancy?	153	29.1%	102	23.2%	0.038*
Was your house renovated before the mother became pregnant?	175	33.3%	112	25.5%	0.008*
Do you use an air conditioner?	450	85.7%	333	75.9%	0.001*
Do you open the windows while sleeping?	144	27.4%	131	29.8%	0.409
Are there cockroaches in your house?	129	24.6%	88	20.0%	0.094
Are there mosquitoes or flies in your house?	261	49.7%	236	53.8%	0.211

Established diagnoses of eczema was reported in 55.8% of children whose mothers cleaned their rooms regularly, compared to 28.3% of those whose mothers cleaned their rooms very rarely (P = 0.001). The climate of the living area had a significant association: 58% of children who live in dry climates had been diagnosed with eczema versus 42% of others who live in wet climates (P = 0.001). Concerning the type of skin, eczema was diagnosed in 70.2% of children with dry skin (P = 0.001) (Table 4).

**Table 4:** Other factors associated with eczema among children in Hail City, Saudi Arabia. P: Pearson X^2^ test; *P < 0.05 (significant).

Other factors	Has a child been diagnosed with eczema?				p-value
	Yes		No		
	No	%	No	%	
What is your house size?					0.086
<75 m^2^	94	46.8%	107	53.2%	
>75 m^2^	431	56.5%	332	43.5%	
How often do you clean the room?					0.001*
Regularly	359	55.8%	284	44.2%	
Occasionally	153	55.6%	122	44.4%	
Very rarely	13	28.3%	33	71.7%	
Have you acquired new furniture in your house during the first year of the child’s/children’s life?					0.713
Yes	215	55.4%	173	44.6%	
No	303	54.2%	256	45.8%	
Have you acquired new furniture in your house during the mother's pregnancy?					0.170
Yes	178	57.8%	130	42.2%	
No	339	53.1%	300	46.9%	
Have you acquired new furniture in your house before the mother became pregnant?					0.573
Yes	176	55.7%	140	44.3%	
No	336	53.8%	289	46.2%	
The climate in the living region?					0.001*
Dry climate	436	58.0%	316	42.0%	
Humid climate	89	42.0%	123	58.0%	
Does your child/children have dry skin?					0.001*
Yes	419	70.2%	178	29.8%	
No	106	28.9%	261	71.1%	
Do you moisturize your child's/children's skin?					0.001*
Yes	446	57.1%	335	42.9%	
No	79	43.2%	104	56.8%	
How often do you moisturize your child's (children's) skin per day?					0.059
1 time	145	49.2%	150	50.8%	
2 times	122	56.2%	95	43.8%	
3 times	96	64.4%	53	35.6%	
4 times	38	69.1%	17	30.9%	
>4 times	45	69.2%	20	30.8%	

The multiple stepwise logistic regression analysis included the variables that were predicted to have a significant impact. Among all the included risk factors, male gender (OR = 1.41, 95%CI = 1.16-1.71), smoking in the house (OR = 1.85, 95%CI = 1.37-2.50), mold or dampness in the house during the mother’s pregnancy (OR = 1.95, 95%CI = 1.17-3.24), use of an air conditioner (OR = 1.57, 95%CI = 1.07-2.30), house renovations during the mother’s pregnancy (OR = 1.54, 95%CI = 1.01-2.34), and dry skin (OR = 5.83, 95%CI = 4.27-7.96) were the most significant risk factors (Table 5).

**Table 5:** Multiple stepwise logistic regression analysis of risk factors for eczema in Hail City, Saudi Arabia. OR_A_: Adjusted odd ratio; CI: confidence interval; *P < 0.05 (significant).

Factors	p-value	OR_A_	95% CI	
			Lower	Upper
Male gender	0.001	1.41	1.16	1.71
Smoking in house	0.001	1.85	1.37	2.50
Mould or dampness in the house while the mother was pregnant	0.010	1.95	1.17	3.24
House renovated during the first year of the child’s/children’s life	0.028	0.65	0.44	0.95
House renovated during the mother's pregnancy	0.045	1.54	1.01	2.34
Use air condition	0.021	1.57	1.07	2.30
Open the windows while sleeping	0.003	0.58	0.41	0.83
Mosquitoes or flies in your house	0.046	0.73	0.54	0.99
Child had dry skin	0.001	5.83	4.27	7.96

## Discussion

Epidemiological studies are extremely crucial in demonstrating the implications of a biological disease in different demographics and populations. Furthermore, highlighting the prevalence and incidence of a particular disease is critical for allocating resources to enhance the level of patient care and evidence-based research. Pediatric population-based epidemiological studies on the prevalence of eczema in Saudi Arabia are scarce and limited. To the best of our knowledge, this is the first study that has investigated the prevalence of eczema and its influencing risk factors among preschool children in Hail City, Saudi Arabia.

Another point to consider is that, according to a recent systematic review, eczema was found to be highly prevalent in Saudi Arabia, with a prevalence of 24% overall from 29,244 dermatologic patients from different regions and age groups. This prevalence was supported by several studies performed in various regions of Saudi Arabia, with a reported prevalence rate ranging from 16% to 37%. Given that climatic and environmental variations may contribute to the disparity in prevalence rates among various regions [12].

In Hail City, no previous study estimated the prevalence of eczema among the preschool population. However, a study conducted in 2017 by Al Shammrie and Al Shammrie on 13,778 patients showed that eczema was the most common skin disorder (37%), with AD accounting for 12% of all eczema cases in Hail City. Additionally, Al Shammrie and Al Shammrie observed that the prevalence of eczema has increased when compared with a previous study by Parthasaradhi et al. [13]. The current study findings showed that 525 (54.5%) parents reported having a child/children with a diagnosed case of eczema. Among these children, 79.1% reported a previous history of an itchy rash that came and went for at least six months, and 78.9% reported symptoms of an itchy rash in the past 12 months. In Urumqi, China, a similar study was conducted by Shi et al., which revealed that 12% of children aged two to eight years reported the confirmed diagnosis of eczema, while 2.3% of children reported symptoms of recurrent rash in the past 12 months, and 7.8% of children reported a previous history of recurrent rash for six months [3]. Locally, Alhazmi et al. conducted a study among schoolchildren in the Jazan region. They reported that 8.9% of their participants had confirmed eczema, 11.4% reported a symptom of an itchy rash that came and went for at least six months, and 8.5% reported a history of the itchy rash in the past 12 months [14]. In another study conducted in Madinah, Nahhas et al. found that 14% of schoolchildren had a diagnosed eczema, 10.4% reported a symptom of an itchy rash for at least six months, and 8.8% reported a history of the itchy rash in the past 12 months [15]. In Najran, a study showed that 12.5% of schoolchildren had a previous diagnosis of AD [16]. A study by Al-Frayh revealed a higher prevalence rate of eczema among children in Hofuf (43.5%) when compared with Riyadh and Jeddah (32.6% and 31.9%, respectively) [17]. Noticeably, the high prevalence rate in the current study when compared to the published literature highlight a real health issue that may require further research and interventions and could be an authentic reflection of the current burden of this dermatological disease among children living in Hail City.

Regarding gender, the present study found a significant difference in having eczema diagnosed between boys and girls. A study by Alqahtani revealed no significant difference in the prevalence of diagnosed eczema between boys and girls. However, the multiple logistic regression analysis identified that male children had a significantly higher risk of developing allergic diseases, which is consistent with the current study [16]. Additionally, Shi et al. found no significant difference between genders [3]. On the other hand, Alhazmi et al. found a statistically significant difference in the prevalence between genders, with a female predominance [14]. According to Govaere et al., male children, particularly those under the age of eight, had significantly higher rates of sensitization to mites, grass, and pollen [18]. Conflicting evidence is existent regarding the role of gender in the prevalence of atopic eczema in children and adolescents [19].

Concerning comorbidities, Ricci et al. conducted a study in Italy that showed that children with eczema have been reported to have rhinitis or asthma at rates as high as 118 cases (57.6%) and 70 cases (34.1%), respectively [20]. In Sweden, 94 children with eczema were followed up to seven years of age, in which allergic rhinitis developed in 45% and asthma developed in 43% [21]. In this study, 74.3%, 72.4%, 75.6%, 75.7%, and 62.0% of children with wheezing chest, asthma, allergic rhinitis, pneumonia, and other comorbidities had concomitant eczema, respectively. Our findings suggest that these comorbidities significantly increase the risk of developing eczema. The concomitance or progressive development of atopic disorders supports the concept of the atopic march [20].

In regards to the mode of delivery, the present study discovered that 57.7% of children with normal vaginal delivery (NVD) had a diagnosed eczema compared to 53.1% of children with cesarean delivery. This contradicts a study by Shi et al., which found that the number of confirmed eczema cases was higher in children delivered via CS than in those with NVD [3]. Several studies have demonstrated that caesarean delivery significantly affects the healthy maturation and development of the neonatal immune system through the disruption of intestinal flora, resulting in immune dysfunction and hyperactive immune responses that could cause allergic reactions [3].

Another aspect to note is that the family history of eczema played a significant role in this study’s findings. Eczema was diagnosed in 76.5% of children with a family history of eczema, compared to 39.3% of other children without a family history. This is consistent with the findings of the study by Shi et al., which established a family history of eczema as a risk factor for eczema development [3]. Furthermore, the development of eczema in infants was also significantly associated with the parental history of atopic diseases, according to earlier studies, which could be explained by the genetic involvement in the disease's pathogenesis [11,22]. Several studies have also suggested that the filaggrin gene (FLG) plays a role in the development of eczema. Given the fact that FLG loss-of-function mutations have been associated with a defect in the epidermal barrier and have been repeatedly implicated as a risk factor for AD [23,24].

Furthermore, the current study found that 70.4% of children with food allergies reported having eczema that had been diagnosed, compared to 43.7% of children without food allergies (P =.001). According to a systematic review, atopic eczema, food sensitization, and food allergy were strongly and dose-dependently associated. Furthermore, several studies [25] discovered that more severe atopic eczema phenotypic expression was associated with a higher rate of food allergy diagnosis.

In our study, we noted that 84.5% of children with pityriasis rosea had eczema. The reason for this is unknown. This was in line with another study that noted that patients with pityriasis rosea and their relatives were reported to have a higher incidence of eczema [26]. We also observed that 75.5% of children with urticaria had eczema. This was in line with observations by Böhme et al., who found a significant association between AD and urticaria in children and that the frequency of urticaria was higher if AD started during the first year of life [27].

Concerning the location of the itchy rash, our study showed that the highest percentage of places affected was the folds of the elbows (41.3%), whereas the vast majority (83.7%) of those affected children had been diagnosed with eczema before. These findings are consistent with a previous study in which the most reported place for eczema among children was the fold of the elbow joint [14].

The present study found that, among multiple environmental risk factors related to a higher prevalence of eczema in preschool children, the first and most common significant risk factor was the presence of carpets in the house. Contrary to these findings, Miyake et al. showed no relationship between atopic eczema and having a carpet in the child's room [28]. Furthermore, human skin is susceptible to mechanical stress after being exposed to a dry environment [29,30]. The second most common risk factor was using an air conditioner at home. This was significantly related to eczema, and similar findings were found in studies carried out by Shi et al. and Wildnauer et al. [3,30]. Additionally, a case-control study conducted in South Korea by Jung et al. showed that the skin barrier of AD patients altered when exposed to cold airflow from the air conditioner, which is considered an aggravating factor of AD [31]. This explains the aggravation of eczema symptoms as a consequence of skin barrier alteration. The third most common risk factor was passive smoking, which was significantly related to eczema in our study, and this was found to be consistent with findings from the studies conducted by Shi et al. and Mitchell et al., in which passive smoking was also found to be linked with an increased risk of eczema [3,32]. Conversely, Tanaka et al. reported no significant relationship between active smoking in a child's bedroom before pregnancy or even during the first year of the baby's life [33], although a prospective cohort study in Denmark reported no statistically significant association between any in utero smoke exposure and the risk of atopic eczema [34]. Similarly, previous epidemiologic studies found no correlation between passive smoking and atopic eczema in children [35-37].

Having household pets was found to significantly increase the risk of developing allergic diseases. Household pets have been recognized in numerous studies as a risk factor for allergic disease development [3,30,38-40]. All these results might be explained by the presence of high levels of allergens in the environment, which leads to increased sensitization and high susceptibility to AD.

Regarding living regions, our study showed that 58% of children who live in dry climates had eczema in comparison to 42% of children who live in humid climates. Alhazmi et al. reported a higher prevalence of AD in students living in plain areas than in coastal and mountain areas [14]. Previous foreign studies found a significant association between country and regional differences and allergic diseases, as the distribution of allergens may vary based on geographic area, local climate, environment, and lifestyle [41,42]. This difference may be related to climatic conditions; a dry climate is suitable for the dispersal of allergens in the environment.

Low humidity affects the skin barrier by increasing the breakdown of filaggrin proteins. These proteins are responsible for skin moisture. Furthermore, filaggrin breakdown increases skin dryness, and then eczema symptoms develop [43]. Furthermore, a human skin study discovered that low climate temperature has a negative impact on the stratum corneum (SC) layer, which protects against excessive water loss, though this relationship is stronger when low temperature is combined with low relative humidity (RH) (60%) [44,45]. RH is explained by the percentage of the actual amount of water vapor in the air divided by the amount of water vapor the air can hold. As a consequence, decreasing SC water content suggests an increased risk of eczema. These findings explain the results shown in the present study, which showed that dry skin was significantly associated with eczema.

The presence of mold or dampness in the house before and during pregnancy and during the first year of the baby’s life was identified as a risk factor for eczema development. A study by Miyake et al. showed a significant positive relationship between mold in the kitchen during pregnancy and the risk of suspected atopic eczema [28]. Additionally, a case-control study in the United Kingdom (UK) found a significant relationship between dampness in the house and atopic eczema [46]. Previous foreign studies also found the same results [47,48].

This study had two potential limitations. First, the study was conducted in Hail City and did not include other cities in Saudi Arabia, which limits the generalizability of our findings to other populations. Second, the questionnaire was filled out by the parents of preschool children, who might have overestimated or underestimated the responses.

## Conclusions

A high prevalence rate of eczema was observed in preschool-aged children in Hail, Saudi Arabia. Male gender, smoking in the house, mold or dampness in the house during the mother’s pregnancy, use of an air conditioner, house renovations during the mother’s pregnancy, and dry skin were considered risk factors for eczema development in preschool children. Thus, the findings of this study emphasize the pressing need for the development of an effective educational program to control and reduce the burden of eczema among children.

## References

[1] Hanifin JM, Reed ML (2007). A population-based survey of eczema prevalence in the United States. Dermatitis.

[2] Odhiambo JA, Williams HC, Clayton TO, Robertson CF, Asher MI (2009). Global variations in prevalence of eczema symptoms in children from ISAAC Phase Three. J Allergy Clin Immunol.

[3] Shi H, Wan G, Wang T (2021). Prevalence and influencing risk factors of eczema among preschool children in Urumqi city: a cross-sectional survey. BMC Pediatr.

[4] Cramer C, Link E, Bauer CP (2011). Association between attendance of day care centres and increased prevalence of eczema in the German birth cohort study LISAplus. Allergy.

[5] Langan SM, Silcocks P, Williams HC (2009). What causes flares of eczema in children?. Br J Dermatol.

[6] Kim HB, Zhou H, Kim JH, Habre R, Bastain TM, Gilliland FD (2016). Lifetime prevalence of childhood eczema and the effect of indoor environmental factors: Analysis in Hispanic and non-Hispanic white children. Allergy Asthma Proc.

[7] Sun C, Zhang J, Huang C (2019). High prevalence of eczema among preschool children related to home renovation in China: A multi-city-based cross-sectional study. Indoor Air.

[8] Sohn A, Frankel A, Patel RV, Goldenberg G (2011). Eczema. Mt Sinai J Med.

[9] Silverberg JI, Hanifin J, Simpson EL (2013). Climatic factors are associated with childhood eczema prevalence in the United States. J Invest Dermatol.

[10] Bornehag CG, Sundell J, Hagerhed-Engman L, Sigsggard T, Janson S, Aberg N (2005). 'Dampness' at home and its association with airway, nose, and skin symptoms among 10,851 preschool children in Sweden: a cross-sectional study. Indoor Air.

[11] Parazzini F, Cipriani S, Zinetti C (2014). Perinatal factors and the risk of atopic dermatitis: a cohort study. Pediatr Allergy Immunol.

[12] Almohideb M (2020). Epidemiological patterns of skin disease in Saudi Arabia: a systematic review and meta-analysis. Dermatol Res Pract.

[13] Al Shammrie F, Al Shammrie A (2017). Pattern of skin disease in Hail region of Saudi Arabia. J Dermatology Dermatologic Surg.

[14] Alhazmi M, Basudan A, Moafa A (2017). Epidemiology of atopic dermatitis among children in Jazan Region, Saudi Arabia. Int J Med Health Res.

[15] Nahhas M, Bhopal R, Anandan C, Elton R, Sheikh A (2012). Prevalence of allergic disorders among primary school-aged children in Madinah, Saudi Arabia: two-stage cross-sectional survey. PLoS One.

[16] Alqahtani JM (2016). Asthma and other allergic diseases among Saudi schoolchildren in Najran: the need for a comprehensive intervention program. Ann Saudi Med.

[17] Al Frayh AS (2005). A 17 year trend for the prevalence of asthma and allergic diseases among children in Saudi Arabia. J Allergy Clin Immunol.

[18] Govaere E, Van Gysel D, Massa G, Verhamme KM, Doli E, De Baets F (2007). The influence of age and gender on sensitization to aero-allergens. Pediatr Allergy Immunol.

[19] Chen W, Mempel M, Schober W, Behrendt H, Ring J (2008). Gender difference, sex hormones, and immediate type hypersensitivity reactions. Allergy.

[20] Ricci G, Patrizi A, Baldi E, Menna G, Tabanelli M, Masi M (2006). Long-term follow-up of atopic dermatitis: retrospective analysis of related risk factors and association with concomitant allergic diseases. J Am Acad Dermatol.

[21] Gustafsson D, Sjöberg O, Foucard T (2000). Development of allergies and asthma in infants and young children with atopic dermatitis--a prospective follow-up to 7 years of age. Allergy.

[22] O'Donovan SM, O'B Hourihane J, Murray DM (2016). Neonatal adiposity increases the risk of atopic dermatitis during the first year of life. J Allergy Clin Immunol.

[23] Riethmuller C, McAleer MA, Koppes SA (2015). Filaggrin breakdown products determine corneocyte conformation in patients with atopic dermatitis. J Allergy Clin Immunol.

[24] Wang XW, Wang JJ, Gutowska-Owsiak D (2017). Deficiency of filaggrin regulates endogenous cysteine protease activity, leading to impaired skin barrier function. Clin Exp Dermatol.

[25] Tsakok T, Marrs T, Mohsin M, Baron S, du Toit G, Till S, Flohr C (2016). Does atopic dermatitis cause food allergy? A systematic review. J Allergy Clin Immunol.

[26] Björnberg A, Hellgren L (1962). Pityriasis rosea. A statistical, clinical, and laboratory investigation of 826 patients and matched healthy controls. Acta Derm Venereol Suppl (Stockh).

[27] Böhme M, Lannerö E, Wickman M, Nordvall SL, Wahlgren CF (2002). Atopic dermatitis and concomitant disease patterns in children up to two years of age. Acta Derm Venereol.

[28] Miyake Y, Ohya Y, Tanaka K (2007). Home environment and suspected atopic eczema in Japanese infants: the Osaka Maternal and Child Health Study. Pediatr Allergy Immunol.

[29] Engebretsen KA, Johansen JD, Kezic S, Linneberg A, Thyssen JP (2016). The effect of environmental humidity and temperature on skin barrier function and dermatitis. J Eur Acad Dermatol Venereol.

[30] Wildnauer RH, Bothwell JW, Douglass AB (1971). Stratum corneum biomechanical properties. I. Influence of relative humidity on normal and extracted human stratum corneum. J Invest Dermatol.

[31] Jung M, Kim I, Lee JY (2020). Exposure to cold airflow alters skin pH and epidermal filaggrin degradation products in children with atopic dermatitis. Allergol Int.

[32] Mitchell EA, Beasley R, Keil U, Montefort S, Odhiambo J (2012). The association between tobacco and the risk of asthma, rhinoconjunctivitis and eczema in children and adolescents: analyses from Phase Three of the ISAAC programme. Thorax.

[33] Tanaka K, Miyake Y, Furukawa S, Arakawa M (2017). Pre- and postnatal smoking exposure and risk of atopic eczema in young Japanese children: a prospective prebirth cohort study. Nicotine Tob Res.

[34] Magnusson LL, Olesen AB, Wennborg H, Olsen J (2005). Wheezing, asthma, hayfever, and atopic eczema in childhood following exposure to tobacco smoke in fetal life. Clin Exp Allergy.

[35] Kurosaka F, Nakatani Y, Terada T (2006). Current cat ownership may be associated with the lower prevalence of atopic dermatitis, allergic rhinitis, and Japanese cedar pollinosis in schoolchildren in Himeji, Japan. Pediatr Allergy Immunol.

[36] Schäfer T, Heinrich J, Wjst M, Krause C, Adam H, Ring J, Wichmann HE (1999). Indoor risk factors for atopic eczema in school children from East Germany. Environ Res.

[37] Haileamlak A, Dagoye D, Williams H, Venn AJ, Hubbard R, Britton J, Lewis SA (2005). Early life risk factors for atopic dermatitis in Ethiopian children. J Allergy Clin Immunol.

[38] Sun Y, Hou J, Sheng Y, Kong X, Weschler LB, Sundell J (2019). Modern life makes children allergic. A cross-sectional study: associations of home environment and lifestyles with asthma and allergy among children in Tianjin region, China. Int Arch Occup Environ Health.

[39] Zhao Y, Liu YQ, Liu MM, Wang D, Ren WH, Gao F, Dong GH (2013). Interactive effects of environmental tobacco smoke and pets ownership on respiratory diseases and symptoms in children. Zhonghua Er Ke Za Zhi.

[40] Kramer MS, Matush L, Bogdanovich N, Dahhou M, Platt RW, Mazer B (2009). The low prevalence of allergic disease in Eastern Europe: are risk factors consistent with the hygiene hypothesis?. Clin Exp Allergy.

[41] Ozkaya E, Sogut A, Küçükkoç M, Eres M, Acemoglu H, Yuksel H, Murat N (2015). Sensitization pattern of inhalant allergens in children with asthma who are living different altitudes in Turkey. Int J Biometeorol.

[42] Migueres M, Dávila I, Frati F (2014). Types of sensitization to aeroallergens: definitions, prevalences and impact on the diagnosis and treatment of allergic respiratory disease. Clin Transl Allergy.

[43] Scott IR, Harding CR (1986). Filaggrin breakdown to water binding compounds during development of the rat stratum corneum is controlled by the water activity of the environment. Dev Biol.

[44] Angelova-Fischer I, Dapic I, Hoek AK, Jakasa I, Fischer TW, Zillikens D, Kezic S (2014). Skin barrier integrity and natural moisturising factor levels after cumulative dermal exposure to alkaline agents in atopic dermatitis. Acta Derm Venereol.

[45] Candi E, Schmidt R, Melino G (2005). The cornified envelope: a model of cell death in the skin. Nat Rev Mol Cell Biol.

[46] McNally NJ, Williams HC, Phillips DR (2001). Atopic eczema and the home environment. Br J Dermatol.

[47] Williamson IJ, Martin CJ, McGill G, Monie RD, Fennerty AG (1997). Damp housing and asthma: a case-control study. Thorax.

[48] Verhoeff AP, van Strien RT, van Wijnen JH, Brunekreef B (1995). Damp housing and childhood respiratory symptoms: the role of sensitization to dust mites and molds. Am J Epidemiol.

